# Innate immunity activation in the early brain injury period following subarachnoid hemorrhage

**DOI:** 10.1186/s12974-019-1629-7

**Published:** 2019-12-04

**Authors:** Typhaine Gris, Patrick Laplante, Paméla Thebault, Romain Cayrol, Ahmed Najjar, Benjamin Joannette-Pilon, Frédéric Brillant-Marquis, Elsa Magro, Shane W. English, Réjean Lapointe, Michel Bojanowski, Charles L. Francoeur, Jean-François Cailhier, on behalf of the Canadian Critical Care Translational Biology Group

**Affiliations:** 1 Research Centre of Centre Hospitalier de l’Université de Montréal (CRCHUM), Montreal, Quebec Canada; 2 CRCHUM and Montreal Cancer Institute, 900 rue St-Denis, Montreal, Quebec, H2X 0A9 Canada; 3 Department of Pathology and Cellular Biology, Faculty of Medicine, Université de Montréal, Pavillon Roger-Gaudry, 5e étage, 2900, Boulevard Édouard-Montpetit, Montreal, Quebec, Canada; 4 Department of Surgery, Division of Neurosurgery, Centre Hospitalier de l’Université de Montréal (CHUM), 850 rue St-Denis, Montreal, Quebec, H2X 0A9 Canada; 5 Neurosurgery Service of CHU Cavale Blanche, INSERM, Boulevard Tanguy Prigent, Finistère, 29200 Brest, Bretagne France; 6 Clinical Epidemiology Program, Ottawa Hospital Research Institute, Civic Campus, 1053 Carling Avenue, Ottawa, ON K1Y 4E9 Canada; 7 Departments of Medicine (Critical Care) and School of Epidemiology and Public Health, Division of Critical Care, The Ottawa Hospital, University of Ottawa, Civic Campus, 1053 Carling Avenue, Ottawa, ON K1Y 4E9 Canada; 8 Population Health and Optimal Health Practices Research Unit (Trauma–Emergency–Critical Care Medicine) and Department of Anesthesiology and Critical Care, CHU de Québec–Université Laval, (Hôpital de l’Enfant-Jésus), 1401, 18e rue, Room Z-204, Québec, G1J 1Z4 Canada; 9 Nephrology Division, CHUM and Department of Medicine, Université de Montréal, Montreal, Quebec Canada

**Keywords:** Cerebral hemorrhage, Neuroinflammation, Innate immunity, Early brain injury, Neuronal death

## Abstract

**Background:**

Aneurysmal subarachnoid hemorrhage (SAH) is a catastrophic disease with devastating consequences, including a high mortality rate and severe disabilities among survivors. Inflammation is induced following SAH, but the exact role and phenotype of innate immune cells remain poorly characterized. We investigated the inflammatory components of the early brain injury in an animal model and in SAH patients.

**Method:**

SAH was induced through injection of blood in the subarachnoid space of C57Bl/6 J wild-type mice. Prospective blood collections were obtained at 12 h, days 1, 2, and 7 to evaluate the systemic inflammatory consequences of SAH by flow cytometry and enzyme-linked immunosorbent-assay (ELISA). Brains were collected, enzymatically digested, or fixed to characterize infiltrating inflammatory cells and neuronal death using flow cytometry and immunofluorescence. Phenotypic evaluation was performed at day 7 using the holding time and footprint tests. We then compared the identified inflammatory proteins to the profiles obtained from the plasma of 13 human SAH patients.

**Results:**

Following SAH, systemic IL-6 levels increased rapidly, whereas IL-10 levels were reduced. Neutrophils were increased both in the brain and in the blood reflecting local and peripheral inflammation following SAH. More intracerebral pro-inflammatory monocytes were found at early time points. Astrocyte and microglia activation were also increased, and mice had severe motor deficits, which were associated with an increase in the percentage of caspase-3-positive apoptotic neurons. Similarly, we found that IL-6 levels in patients were rapidly increased following SAH. ICAM-1, bFGF, IL-7, IL-12p40, and MCP-4 variations over time were different between SAH patients with good versus bad outcomes. Moreover, high levels of Flt-1 and VEGF at admission were associated with worse outcomes.

**Conclusion:**

SAH induces an early intracerebral infiltration and peripheral activation of innate immune cells. Furthermore, microglia and astrocytic activation are present at later time points. Our human and mouse data illustrate that SAH is a systemic inflammatory disease and that immune cells represent potential therapeutic targets to help this population of patients in need of new treatments.

## Background

Aneurysmal subarachnoid hemorrhage (SAH) is a catastrophic disease associated with significant mortality and morbidity in patients. Approximately 35% of patients will die during the first 30 days following SAH [1]. Unlike other forms of stroke, SAH affects a predominantly young population (40-60 years) [2]. Given its high mortality and morbidity [3], SAH is a significant cause of premature death and loss of potential life years [4, 5]. Among survivors of the initial bleed, an as-yet-imprecise secondary brain injury is the cause of major morbidity [6]. Historically, this mortality and morbidity were thought to be predominantly related to brain edema and/or arterial vasospasm [2, 7, 8]. However, studies have shown that despite reduction of SAH incidence or targeted therapies, patient outcomes have not change [6]. A recent study from Nassiri et al., through a propensity score-matched analysis from the Clazosentan to Overcome Neurological iSChemia and Infarction OccUring after Subarachnoid hemorrhage (CONSCIOUS) study, showed beneficial effect of non-steroidal anti-inflammatory drugs on mortality in SAH patients without any difference on vasospasm and delayed cerebral ischemia (DCI) [9]. It is clear that events in the first 72 h after SAH, the early brain injury (EBI) period, are crucial to the viability of neurons, the loss of which is complicit with the altered functional status of these patients [10]. The exact nature of EBI in SAH is ill-defined, but inflammation following SAH represents a pathway of great interest. Multiple studies have associated high cytokine levels with bad outcomes in SAH patients [6, 10]. Innate cells have been inconsistently involved in the immune response following SAH [11–15]. Altogether, these studies suggest that cellular inflammation is activated during EBI and may be important in DCI. However, there is a gap in the comprehensive appreciation of the role of inflammation in neuronal cell death after SAH. Our objectives were to characterize the inflammatory-like events (referred to as inflammation from hereon) during EBI and the associated neuronal cell death and motor functions in a mouse model of induced SAH by the following [1]: Measuring the blood cytokine response and leukocyte activation, brain monocytes, neutrophil infiltration, and microglia and astrocytic activation at multiple time points following SAH [2]; assess caspase-3 activation and cell death in neurons; and [3] assess motor function. We also compared measures of the blood cytokine response in our murine model of SAH to blood inflammatory protein profiles in adult patients with SAH in a prospectively enrolled cohort from our critical care unit.

## Methods

### Mouse model of SAH

We used adult male C57BL/6 J wild-type (WT) mice between 10-12 weeks old (n = 125). Mice were housed with water and food *ad libitum*. We used an adapted SAH model [16] that was approved by the Comité Institutionnel de Protection des Animaux (CIPA) of the Centre de Recherche du Centre Hospitalier de l’Université de Montréal (CRCHUM). In brief, the day before surgery, mice received acetaminophen (160 mg/125 mL H_2_O) diluted in water. After anesthesia (isofluorane 2%), the head was fixed on a stereotactic frame (Stoelting, Woo Dale, IL). Following incision of the scalp, a 0.8 mm diameter hole was drilled (CircuitMedic, Haverhill, MA) through the skullcap, at 2 mm anterior to the bregma at a 40° angle. To induce SAH in mice (n = 60), 100 μL of isogenic blood from a second mouse (obtained through intracardiac puncture) was injected into the pre-chiasmatic cisternae of the recipient using a 27G spinal needle (Braun medical, Bethlehem, PA). Needle insertion without blood injection served as control (SHAM, n = 59). The normal group (n = 6) were unmanipulated mice. Mice were randomly divided into each group.

### Cytokine measurements

The U-Plex technology from Mesoscale Discovery (MSD, Rockville, MD) was used to measure cytokine levels in mouse plasma samples (nSHAM = 19, nSAH = 20). The kit selected (interleukin (IL)-1β, IL-6, IL-10, IL-17A, IL-23, and tumor necrosis factor (TNF)-α) was used per manufacturer’s instructions.

### Flow cytometry

#### Blood cells

**Fig. 1:**
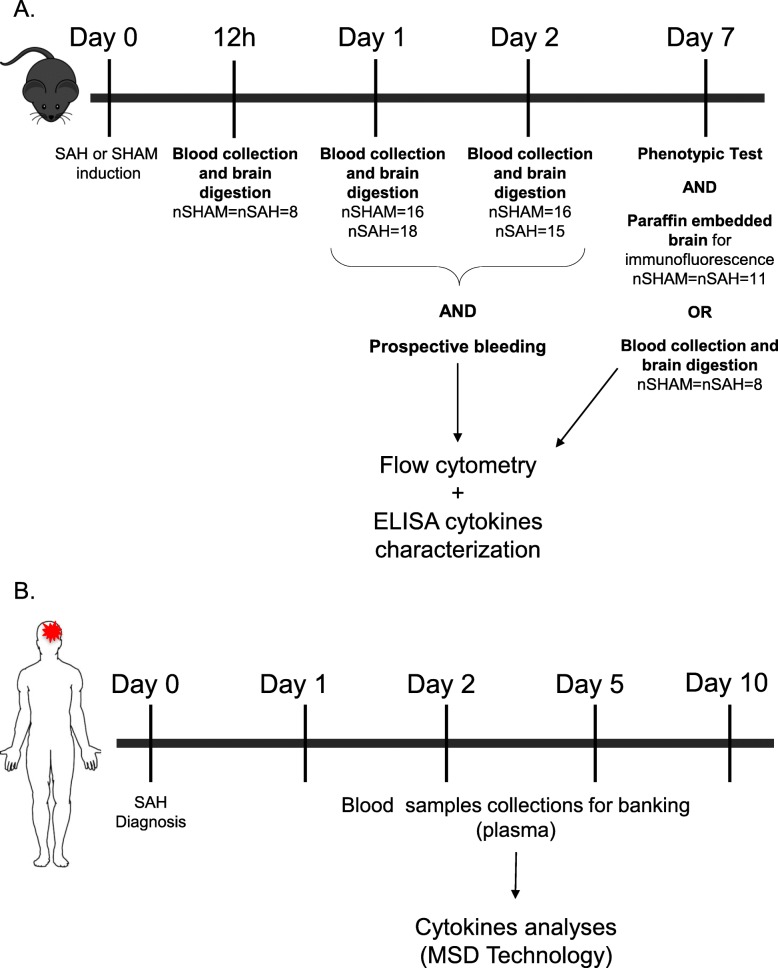
Experimental design. **a** Experimental design for mice experiments. SAH was induced with surgery at D0. At 12 h (nSHAM = nSAH = 8), the mice were sacrificed, and brain and blood were prepared for flow cytometry analyses. At D1 blood was collected by saphenous bleeding for flow cytometry (nSHAM = 35, nSAH = 34) or the mice were sacrificed, and brain and blood were prepared for flow cytometry analyses (nSHAM = 16, nSAH = 18). At D2, blood was collected by saphenous bleeding for flow cytometry (nSHAM = nSAH = 19) or the mice were sacrificed, and brain and blood were prepared for flow cytometry analyses (nSHAM = 16, nSAH = 15). At D7, the phenotypic tests were performed (nSHAM = nSAH = 19) and blood and the brains were conserved, and paraffin embedded for immunofluorescence essays (nSHAM = nSAH = 11) or brains were prepared for flow cytometry (nSHAM = nSAH = 8). **b** Experimental design for human immunomonitoring. At D0, SAH patients were recruited within the first 48 h. At D0, D1, D2, D5, and D10, blood was collected and centrifuged for banking plasma. Plasma were used for cytokine analyses by MSD technology

Blood was collected by prospective saphenous bleeding (D1 (nSHAM = 35, nSAH = 34, 8 independent experiments) and D2 (nSHAM = nSAH = 19, 4 independent experiments)] or by terminal intracardiac puncture (12 h (nSHAM = nSAH = 8, 2 independent experiments), D1 (nSHAM = 16 nSAH = 18, 4 independent experiments), D2 (nSHAM = 16, nSAH = 15, 4 independant experiments), and D7 (nSHAM = nSAH = 8, 2 independent experiments)) (Fig. 1a), then centrifuged (4000 rotations per minutes (rpm), 15 min, 4 °C) to collect plasma, which was used for cytokine measurements. Red blood cells were eliminated following lysis with Ammonium Chloride Potassium buffer (ACK: 1.5 M NH_4_Cl, 100 mM KHCO_3_ and 100 mM ethylenediaminetetraeacetic acid (EDTA)) for 5 min with gentle shaking at 4 °C. Cells were then centrifuged, washed with FACS buffer (phosphate-buffered saline (PBS), 2% fetal bovine serum, 5 mM EDTA) and subjected to staining. Blood was taken from SAH, SHAM, and unmanipulated mice.

#### Brain cells

At several time points (12 h, D0, D1, D2, and D7) after SAH or SHAM induction or for the unmanipulated group (Fig. 1a), mice were anesthetized, and the vasculature was flushed by intracardiac injection of 25 mL of saline solution (0.9% NaCl). Brains were subsequently harvested, sliced with a scalpel, and enzymatically digested (2 mg/mL of collagenase D and 14 μg/mL of DNase) for 15 min at 37 °C, and finally filtered through a 100 μm mesh. After a wash with Hank’s balanced salt solution (HBSS) (Gibco, Hampton, NH) and centrifugation (1400 rpm, 7 min, 4 °C), the myelin was removed with a 37% Percoll solution (GE Healthcare Bio-science, Uppsala, Sweden; diluted with HBSS) and brain cells were washed with HBSS before staining [17].

#### Cell staining

Cells were first blocked with purified rat anti-mouse C16/CD32 Fc block (BD Pharmingen, San Jose, CA) for 30 min at 4 °C. After a wash with FACS buffer, cell surface staining was performed at 4 °C for 30 min using the following markers: monoclonal rat anti-mouse CD45 PeCy7 (1/500, eBioscience, San Diego, CA), rat anti-mouse Ly6C BV421 (1/800, BD Horizon, Mississauga, ON, Canada), rat anti-mouse Ly6G FITC (1/1000, BD Pharmingen), and rat anti-mouse CD11b AF700 (1/400, BD Pharmingen). Cell viability was assessed using the Live/Dead Fixable Aqua Dead Cell Stain Kit (Invitrogen, Carlsbad, CA). Stained cells were acquired on a LSR Fortessa cytometer (BD Immunocytometry Systems, San Jose, CA) and data collection was obtained using the BD FACS Diva software (BD Bioscience, Mississauga, Ontario, Canada). Data analyses were performed using the Flowjo software (v.10.2, flowjo, LLC).

### Immunofluorescence analysis

At D7 (Fig. 1a), mice (nSAH = nSHAM = 11, 2 independent experiments and nNormal = 3) were anesthetized and the vasculature was flushed by intracardiac injection of 25 mL of saline solution (0.9% NaCl) and 25 mL of formalin. Formalin-fixed paraffin-embedded brains were sectioned transversally in 6-μm slices and stained manually. After deparaffinization in three successive 5-min baths of xylene, and rehydration in an ethanol gradient (95%, 70%, 30%), antigen retrieval was achieved with an EDTA buffer (1 mM EDTA, 0.05% Tween-20 adjusted to pH 8.0) for 20 min at boiling temperature and then washed for 15 min in running water. Tissues were then permeabilized with 0.25% Triton X-100 in PBS for 30 min at room temperature and washed three times (5 min each) in PBS. The sections were blocked with a BlockAid solution from Life Technologies (Carlsbad, CA) for 1 h at room temperature, washed, and incubated at 4 °C overnight with the following antibodies: mouse monoclonal anti-mouse Neuronal nuclei (NeuN) antibody (Millipore, Billerica, MA) and rabbit anti-mouse cleaved caspase-3 (Asp175) antibody (Cell Signaling, Danvers, MA) or rabbit anti-mouse ionized calcium-binding adapter molecule 1 (Iba-1) antibody (Wako chemicals, Richmond, VA), and mouse monoclonal anti-mouse glial fibrillary acidic protein (GFAP)-Cy3 antibody (Sigma-Aldrich, Saint-Louis, MO). After washes, sections were incubated for 1 h with their respective secondary antibodies (either anti-rabbit Alexa-647 or anti-mouse Alexa-594) from Life Technologies before counterstaining using ProLong® Gold Antifade Reagent with DAPI (Molecular Probes, Eugene, OR). Substitution of the primary antibody served as negative control. A Zeiss Observer Z1 fluorescent microscope with the AxioVision rel4.8 program (Zeiss, Oberkochen, Germany) was used to read the slides in a blinded fashion. For the astrocytes (GFAP) and microglia (Iba-1) quantification, three pictures (20x) around each ventricle were taken, and the positive pixels were measured using the imageJ software (v 1.6.0). For apoptotic neuron quantification (NeuN and cleaved caspase-3), six random pictures (× 20) were taken throughout the whole section. As suggested in the literature [18], two blinded investigators manually determined co-localized signals and an average was obtained per mice.

### Phenotype evaluation

Motor capacities were evaluated at D7 (nSHAM = nSAH = 19, 4 independent experiments and nNormal = 3) (Fig. 1a) in order to confirm SAH induction using two phenotypic tests: the holding time test and the footprint test. The evaluating investigator was blinded to the experimental conditions. The holding time test is adapted from the inverted grid test [19]. Briefly, a cotton-tipped applicator (Fisher scientific, Hampton, NH) was placed and fixed on a pedestal at a 30° angle. Mice were then placed on it and the time during which the mouse stayed suspended was measured. Measures were done in triplicate per mouse to obtain an average time. For the footprint test [20], front and rear paws were respectively colored with yellow and blue nontoxic paint. Animals were then allowed to freely walk on a white paper sheet and only the regions in which the mice walked in a straight line were considered for the gait quantification. Gait pattern was analyzed by a scoring system based on a normal mouse behavior. The score of 0 was given when front and rear paws reached the same level (superposition) while walking. The score of -1 was given when rear paws, left and/or right, were not able to match the position of the front paws (no superposition) while walking, hence reflecting a motor deficit phenotype. Three measurements per paw-side per mouse were obtained.

### Human immunomonitoring

We recruited consecutive adult patients who had a diagnosis of SAH in the last 48 h and were admitted to an academic intensive care unit between May 2013 and March 2015. Diagnosis was made by head computed tomography (CT) or the presence of red blood cells and xanthochromia on cerebrospinal fluid (CSF) analysis. Patients with secondary SAH related to trauma, rupture of an arteriovenous malformation or other structural lesions were excluded, as well as patients with significant immunosuppression/cancers/chronic organ failure/chronic viral infection. Written informed consent was obtained from the patient or a legally authorized representative prior to their inclusion in the study. The study was approved by the CHUM ethics committee.

All patients were admitted to a critical care unit and treated as per contemporary clinical guidelines [21]. They received 60 mg per os nimodipine every 4 h and surgical or endovascular treatment was performed as soon as possible. External ventricular drainage (EVD) was inserted as needed, for symptomatic hydrocephalus or intraventricular hemorrhage with reduced level of consciousness. Blood samples were taken at admission and at D1, D2, D5, and D10 (Fig. 1b). Plasma was obtained after centrifugation and frozen until completion of all time points. The V-Plex technology from MSD was used to measure cytokine levels in plasma. The kit selected (c-reactive protein (CRP), Eotaxin, Eotaxin-3, basic fibroblast growth factor (bFGF), granulocyte-macrophage colony-stimulating factor (GM-CSF), intercellular adhesion molecule (ICAM-1), interferon-γ (IFN-γ), IL-10, IL-12/IL-23p40, IL-12p70, IL-13, IL-15, IL-16, IL-17A, IL-1α, IL-1β, IL-2, IL-4, IL-5, IL-6, IL-7, IL-8, interferon-inducible protein (IP)-10, monocyte chemoattractant protein-1 (MCP-1), MCP-4, macrophage-derived chemokine (MDC), macrophage inflammatory protein (MIP)-1α, MIP-1β, placental growth factor (PlGF), serum amyloid A (SAA), thymus and activation-regulated chemokine (TARC), tyrosine kinase with immunoglobulin and EGF homology domains (Tie)-2, TNF-α, TNF-β, vascular cell adhesion molecule (VCAM)-1, vascular endothelial growth factor (VEGF)-A, VEGF-C, VEGF-D, VEGFR-1/Flt-1)) was used per manufacturer’s instructions. Clinical outcome was evaluated at one year using the modified Rankin Scale (mRS). This mRS is a clinical severity scale based on the patient’s neurological disabilities [22].

### Statistical analysis

The results are expressed as mean +/− standard error of the mean (SEM) and were analyzed by Student’s *t* test (with Bonferroni correction when appropriate). *P* < 0.05 was deemed to be significant for all tests. For human cytokine analyses, within-group analysis with repeated measure ANOVA was used to confirm significant variation in levels of blood-derived inflammatory factors across time in individual patients. Mean levels of inflammatory mediators stratified by dichotomized outcomes (good [mRS 0–3] or poor [mRS 4–6]) were plotted against time with their 95% confidence interval (CI) using the groupwiseMean function from the rcompanion package in R. Ordinal logistic regressions were used to model the association between each serum-derived inflammatory mediator at admission and clinical outcomes. The roportional odds model was used and was fitted with the “polr” function from the MASS package in R, with 95% CI. All analyses were performed using R version 3.3.1.

## Results

### SAH induces a peripheral and systemic inflammatory response

**Fig. 2:**
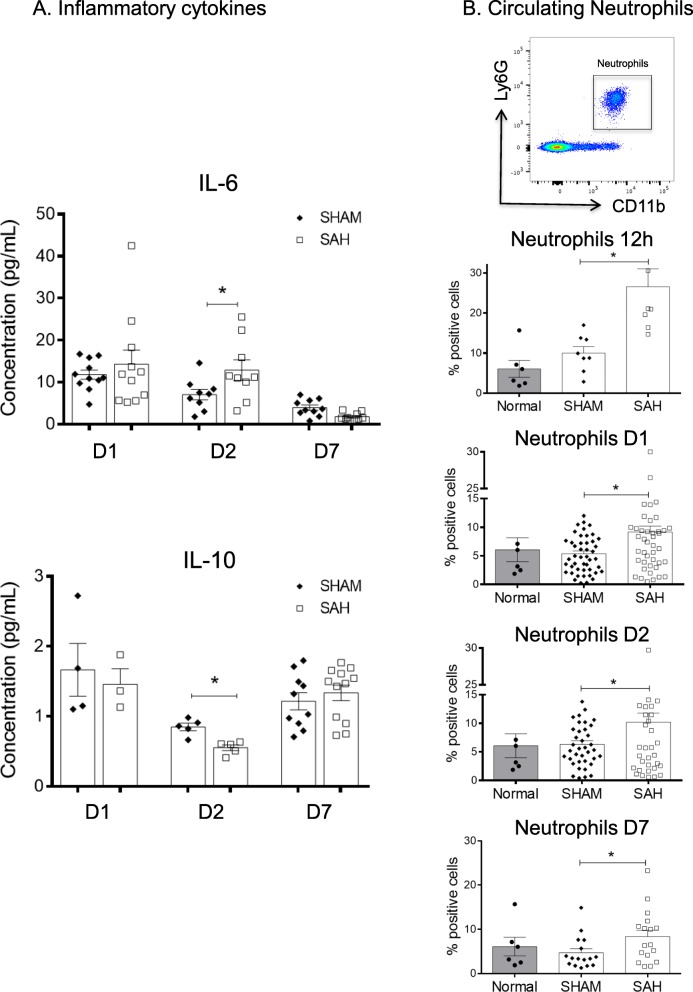
SAH is linked to a systemic modulation of inflammatory cytokines and neutrophils. **a** Quantification of IL-6 and IL-10 levels in the plasma at D1 (nSHAM = 4, nSAH = 3), D2 (nSHAM = nSAH = 5) and D7 (nSHAM = 10, nSAH = 12). At D2, we found a significant increase in IL-6 (*p = 0.049) and significant decrease in IL-10 (*p = 0.002) levels in the SAH mice (white squares) compared with SHAM mice (black diamonds). **b** Representative dot plots and quantification of neutrophil percentages (CD11b^+^Ly6G^+^) in the blood. Over time, we observed a sustained increase in neutrophil percentages at 12 h (*p = 0.0035, nSHAM = nSAH = 8), D1 (*p = 0.002, nSHAM = 51, nSAH = 52), D2 (*p = 0.027, nSHAM = 35, nSAH = 34) and D7 (*p = 0.042, nSHAM = nSAH = 19) following SAH (white squares) compared with SHAM (black diamonds). The neutrophil percentages from normal mice (*n* = 6, black circles) are not different from the SHAM mice

To decipher the early inflammatory innate response present in our model, we first characterized the activation of peripheral cellular inflammation after SAH with prospective blood sample analyses. At 24 h, we observed an increase in pro-inflammatory IL-6 levels and a decrease in anti-inflammatory IL-10 levels. At 48 h, this trend was confirmed by a significant increase in pro-inflammatory IL-6 levels and a significant decrease in anti-inflammatory IL-10 levels (Fig. 2a). According to the published literature [23, 24], the IL-6 and IL-10 values of adult male C57BL/6 J WT are in the expected range of normal mice. However, no variations were observed in levels of IL-17, IL-23, TNF, or IL-1β. SAH also induced a persistent and significant systemic neutrophilia (CD11b^+^Ly6G^+)^ for all the studied time points (Fig. 2b). The blood monocyte subset populations were not modulated following SAH (data not shown). Our analyses support higher levels of systemic inflammatory mediators in the SAH group when compared with the SHAM group.

### SAH induces early intracerebral innate immune cell recruitment and activation

**Fig. 3:**
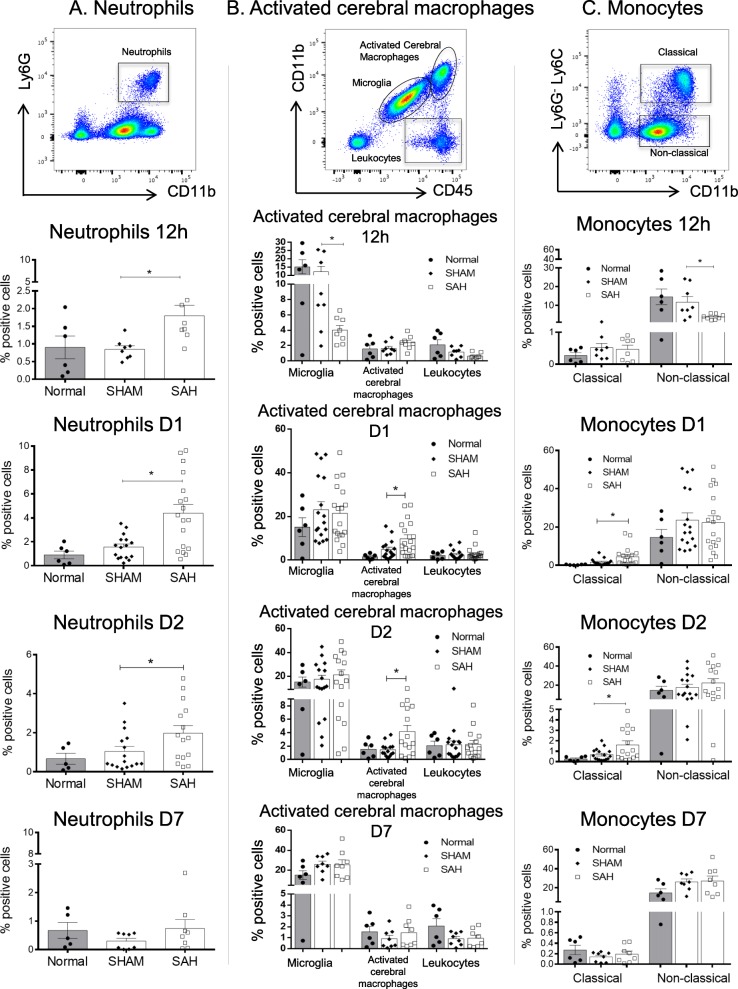
SAH induction was locally associated with activation of microglia and pro-inflammatory monocytes, and neutrophil recruitment. Flow cytometry analyses of brain infiltrating leukocytes. **a** Representative dot plot for quantification of neutrophil percentages (CD11b^+^Ly6G^+^) at 12 h (nSHAM = nSAH = 8), D1 (nSHAM = 16, nSAH = 18), D2 (nSHAM = 16, nSAH = 15) and D7 (nSHAM = nSAH = 8). We observed a significant increase in neutrophils following SAH compared with SHAM at 12 h (*p = 0.0080), D1 (*p = 0,030) and D2 (*p = 0.017). Neutrophils were almost undetectable at D7. Values from the normal group showed no significant differences between normal and SHAM groups. **b** Representative dot plot and quantification of positive cell percentages of microglia (CD45^low^CD11b^low^), activated microglia and macrophages (CD45^high^CD11b^high^) and leukocytes (CD45^+^CD11b^-^) at 12 h, D1, D2, and D7. We observed a significant decrease in microglia at 12 h (*p = 0.0286) and a significant increase in activated microglia at D1 (*p = 0.031) and D2 (*p = 0.0085) following SAH compared with SHAM. No significant differences were observed for microglia and leukocytes over time using this strategy. No significant differences were observed between normal and SHAM groups. **c** Representative dot plot and quantification of positive cell percentages for pro-inflammatory (Ly6G^-^CD11b^+^Ly6C^high^) and anti-inflammatory (Ly6G^-^CD11b^+^Ly6C^low^) monocytes at 12 h, D1, D2, and D7. We observed a significant decrease in non-classical monocytes at 12 h (*p = 0.0225) followed by a significant increase in pro-inflammatory monocytes at D1 (*p = 0.049) and D2 (*p = 0.020) following SAH compared with SHAM. Monocytes, either classical or non-classical, were almost undetectable at D7. There was no significant difference between normal and SHAM groups

We characterized the brain infiltrating innate immune cells. At 12 h, D1 and D2 after SAH, we highlighted a significant increase in intracerebral neutrophil (CD11b^+^Ly6G^+^) proportions compared with the SHAM group (Fig. 3a). However, this difference was not seen at D7 (Fig. 3a). At D1 and D2, a significant increase in intracerebral macrophages and activated microglia (CD45^high^CD11b^high^) [17] was observed in the SAH group when compared with the SHAM group (Fig. 3b). No difference in resting microglia (CD45^low^CD11b^low^) [17] was detected between the two groups. To further characterize brain innate immune cells, we examined two different monocyte populations: the Ly6G^-^CD11b^+^Ly6C^low^ cells (non-classical or anti-inflammatory monocytes) and Ly6G^-^CD11b^+^Ly6C^high^ cells (classical or inflammatory monocytes) [25]. Interestingly, we observed a significant early decrease in non-classical monocytes at 12 h associated with an increase in classical monocytes at D1 and D2 in the SAH group compared with the SHAM group (Fig. 3c). Taken all together, these results suggest that brain inflammation following SAH is characterized by microglia and macrophage activation, as well as neutrophils and classical monocyte infiltration.

### SAH promotes microglia and astrocyte activation and neuron apoptosis

**Fig. 4:**
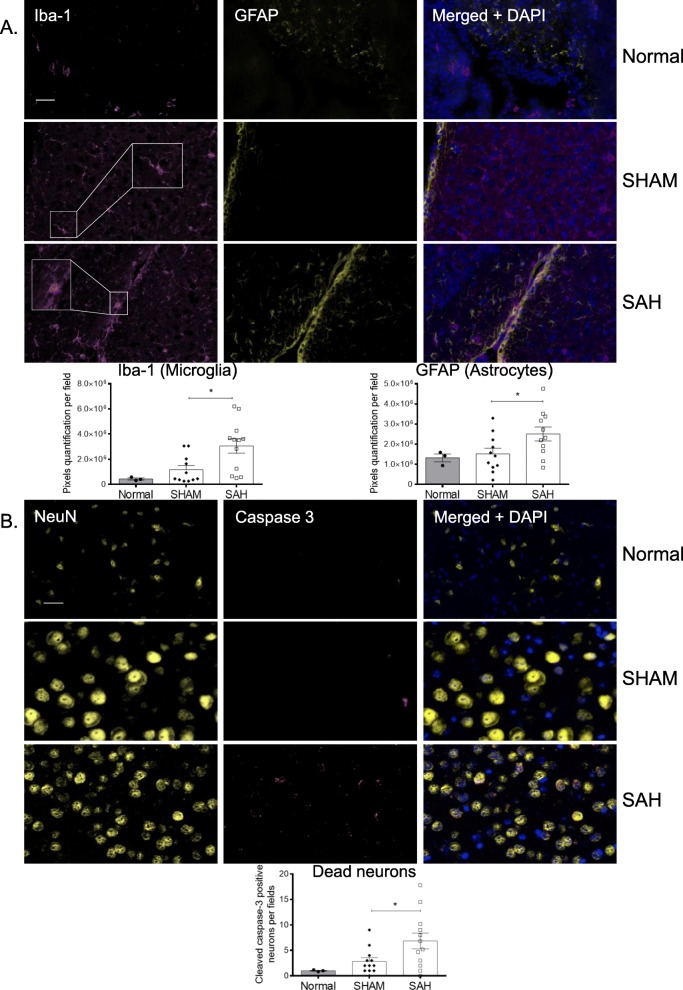
SAH induces a local activation of microglia and astrocytes, and caspase-3-positive apoptotic neurons. Immunofluorescence staining was performed at D7 on brain slides (nSHAM = nSAH = 11). **a** Representative pictures of activated microglia (Iba-1, pink) and astrocytes (GFAP, yellow) are shown for the different groups (Normal, SHAM, and SAH). Quantification of pixels showed that SAH significantly induced microglia (*p = 0.009) and astrocyte (*p = 0.021) activation compared with control (SHAM). **b** Representative pictures of neurons (NeuN, yellow) and activated caspase-3 (cleaved caspase-3, red) are shown for the different groups (Normal, SHAM, and SAH). Merged images showing colocalization define apoptotic neurons. Quantification showed that SAH induced a significant increase in neuron death compared with control (*p = 0.010). DAPI was used as a counterstain for nuclei (bar = 50 μm).

Iba-1 and GFAP immunofluorescence staining to measure microglia and astrocyte activation respectively is a method commonly used in subarachnoid models [26–28]. We found a significant increase in activated cerebral macrophages and astrocytes in the SAH group compared with the SHAM group at D7 (Fig. 4a). Under simple magnification (Fig. 4a), microglia morphology changes were detected between SHAM and SAH with Iba-1 staining. The majority of Iba-1 positive cells in the SHAM group were similar to resting microglia (ramified morphology) whereas the Iba-1 positive cells in the SAH group appeared to have a larger cell body associated with a decrease of ramification similar to the ameboid (activated) microglia [29]. We also found a significant increase in apoptotic neurons, identified by dual positivity for NeuN and cleaved active caspase-3, in SAH mice compared with SHAM at D1 (data not shown) and D7 (Fig. 4b). These results suggest that SAH induced the activation of microglia and astrocytes and neuronal apoptosis.

### Induction of SAH by blood injection induces motor deficits in mice

**Fig. 5:**
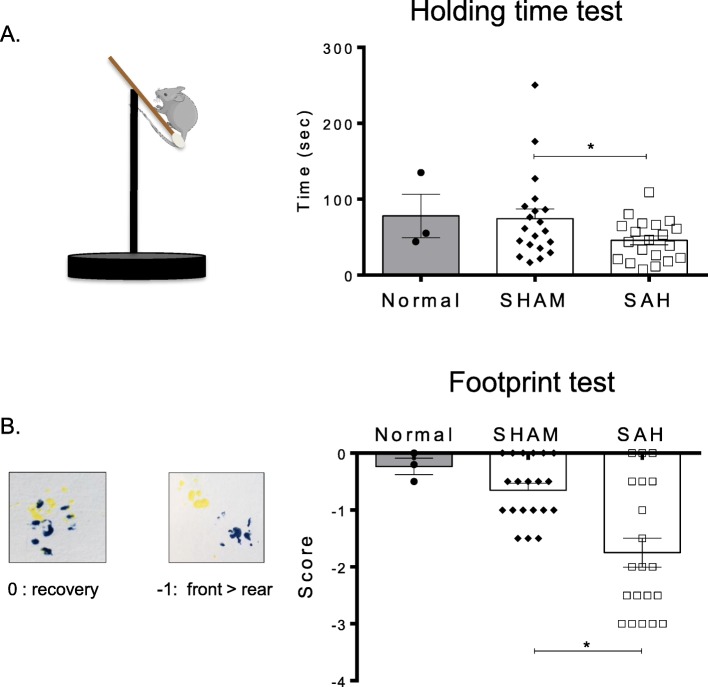
SAH induces motor deficiencies. Tests were performed at D7 on SHAM, SAH mice (nSHAM = nSAH = 19) and 3 normal mice. **a** Holding time test. The SAH group had a significant decrease in its holding capacity compared with the SHAM group (*p = 0.003)**. b** Footprint test. The SAH group showed a significantly deficient gait compared with the SHAM group (*p = 0.001)

To validate the efficiency of our mouse model and to confirm that it induces similar signs to what is observed in human patients during DCI, the presence of motor deficits following SAH was confirmed by two complementary phenotypic tests [19, 20]. First, SAH mice had a significant decrease in their holding time as compared with control (SHAM) mice (Fig. 5a). Secondly, we used the footprint test to demonstrate and quantify motor deficits and observed a poorer performance in SAH mice than in SHAM mice on gait analysis (Fig. 5b).

### Human immunomonitoring in plasma

**Fig. 6:**
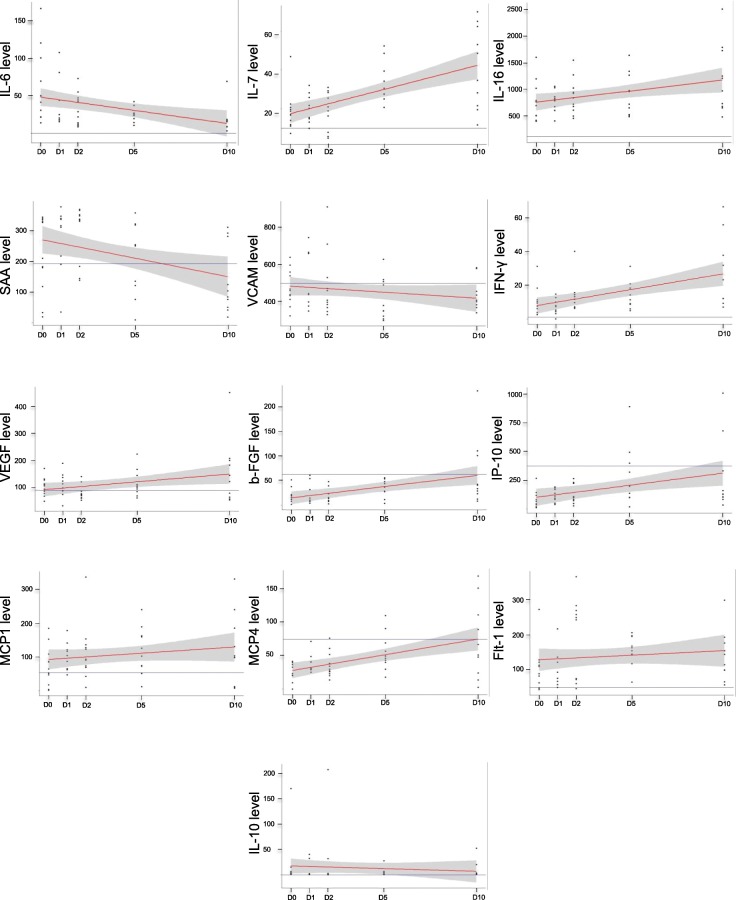
Cytokine variation over time by human immunomonitoring. Mean levels and distribution of selected cytokines at D0, D1, D2, D5, and D10, with baseline levels in healthy controls (line parallel to the *x* axis). We demonstrated a significant variation over time in levels of inflammatory mediators shown in the figure using within-group analysis with repeated measure ANOVA (*p* value< 0.05)

**Fig. 7:**
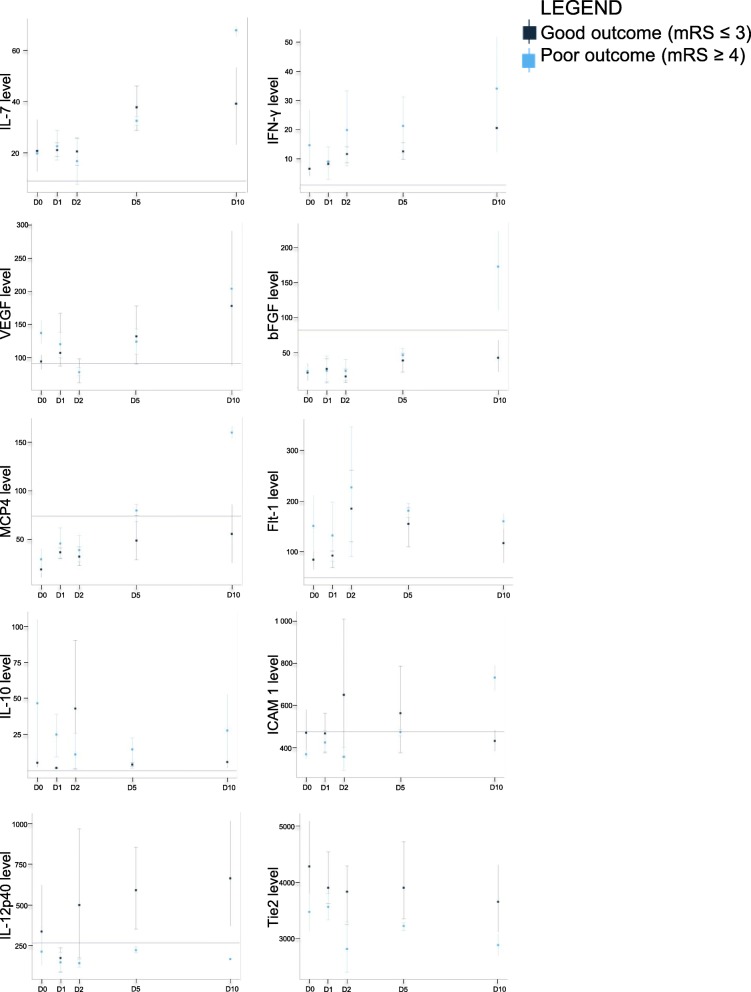
Cytokine variation and outcomes by human immunomonitoring. Group-wise mean levels and distribution of selected cytokines at D0, D1, D2, D5, and D10 stratified by outcomes, 7 patient had good outcomes (good outcome defined as an mRS of 0–3 at 1 year), 3 patients had worse outcomes and 3 patients had unknown outcomes, with baseline levels in healthy controls (line parallel to the *x* axis). Our observations support a different longitudinal evolution among patients with favorable outcomes compared with those with a worse outcome based on ICAM-1, bFGF, IL-7, IL-12p40, and MCP-4

A total of 13 adult patients with SAH were recruited, 86% of which presented with significant brain injury (high-grade SAH with World Federation of Neurological Surgeons (WFNS) grade 4 or 5 at admission) (see Table 1 for patient characteristics). Good neurological outcome was observed in 43% at 12 months (mRS 0–3). Levels of IL-6, IL-7, IL-16, SAA, VCAM-1, IFN-γ, VEGF, bFGF, IP-10, MCP-1, MCP-4, and Flt-1 all varied significantly in time (Fig. 6). The longitudinal variations of inflammatory mediators were markedly different among patients with favorable outcomes compared with those with a worse outcome in regard to ICAM-1, bFGF, IL-7, IL-12p40, and MCP-4 (Fig. 7). Despite the small number of patients, admission levels of blood inflammatory factors showed trends of association with outcome (data not shown). Based on ordinal logistic regression, higher levels of Flt-1 and VEGF at admission were significantly associated with worse outcomes (odds ratio 1.23 [95% C.I. 1.004, 1.586] and 1.70 [95% C.I. 1.05, 3.42] respectively). However, wide confidence intervals with most of the other inflammatory mediators prevented meaningful conclusions.

**Table 1:** Characteristics of adult patients with SAH

Patient	Sex	Age	WFNS grade	Fisher grade	mRS	Comorbidities
1	F	59	4	4	3	Arterial hypertension, dyslipidemia, appendectomy, tonsillectomy, smoking
2	F	63	2	4	1	Arterial hypertension
3	F	59	5	4	3	Arterial hypertension, anemia
4	F	46	4	4	4	Multiple sclerosis, substance addiction
5	M	51	2	3		Smoking
6	M	51	3	3	1	Smoking
7	M	74	5	3		Parkinson disease
8	M	55	5	4	5	Anxiety, depression, chronic alcoholism
9	M	63	5	4	3	Atherosclerotic heart disease, epilepsy, arterial hypertension, chronic obstructive pulmonary disease
10	F	62	5	4	4	Car-pedestrian accident
11	F	57	5	4		Null
12	F	49	4	4	2	Arterial hypertension, nephretic colic, smoking
13	M	52	5	4	2	Null

*F* female, *M* male, *WFNS* World Federation of Neurological Surgeons, *mRS* modified Rankin Scale

## Discussion

We took advantage of a mouse SAH model that mimics an anterior circulation aneurysm rupture. This model is associated with a prolonged increased in peripheral neutrophil mobilization and a significant increase of IL-6 levels reflecting activation of systemic inflammation. This inflammatory systemic response is translated into an early increase in neutrophil recruitment to the brain (12 h to D2). Following SAH, we also observed an early increase in brain infiltration of innate immune cells. Furthermore, we found an early increase in microglia activation and pro-inflammatory monocyte recruitment within the first 48 h after SAH during the EBI period. Similar to SAH in humans, the presence of apoptotic neurons is associated with motor complications [10] at later time points as confirmed by our two phenotypic tests highlighting motor dysfunctions [30] and muscle weakness [31].

Various events, including brain inflammation as investigated here, occur during EBI and have been implicated in the late increase in neuronal death that is responsible for the development of neurologic symptoms observed in SAH, referred to as DCI [32]. We found significant modulations of IL-6 and IL-10 levels after SAH in our mouse model. In opposition to other studies, we did not find any differences in TNF-α and IL-1β after SAH [33]. In our mice model, the increase of the blood-brain barrier (BBB) permeability [3] following SAH was confirmed indirectly by the significant increase of circulating and brain neutrophils and brain monocytes. Neutrophils have a phagocytic function to eliminate red blood cells [34], but they also release pro-inflammatory factors exacerbating inflammation [35]. The increase of neutrophils in our model and a study that showed that depletion of neutrophils improved survival in rats [36] suggest that they may be important in the neuroinflammation induced after SAH.

In our model, SAH also induced microglia activation, reflecting initiation of cerebral inflammatory events. These results were also observed in other inflammatory models such as spinal cord injury models [37]. Microglial cells play a crucial role in inflammatory processes [38]. Activated microglia can prevent neuronal injury and promote tissue repair, but hyperactivation of microglia can promote cell death and neuronal dysfunctions through high secretion of pro-inflammatory cytokines (TNF-α, IL-1β, IL-6) [39]. The microglia is also the major component of the innate immune system in the brain and is composed of resident macrophages. These macrophages are reprogrammed in response to their microenvironment into several phenotypes ranging from pro-inflammatory to anti-inflammatory macrophages [40]. The intracerebral macrophages are also generated from the differentiation of recruited monocytes into brain macrophages [41]. Our data suggest that SAH induced a significant increase in activated resident microglia and recruited classical monocytes at D1 and D2. Hence, we believe that monocytes are activated and differentiated locally in response to SAH. Astrocyte activation, as observed in our model, is probably a consequence of microglia activation, enhancing neuroinflammation [42]. Moreover, early microglial and astrocyte activation results in macrophage activation through monocyte [43] and neutrophil [34] recruitment, which enters the subarachnoid space early after SAH to activate and maintain local cellular inflammation.

Neuron apoptosis has also been detected after SAH in humans [44] and in animal models [45]. Cleaved caspase-3 is used as a marker of apoptotic cell death because of its effector function [46, 47]. We showed that the induction of SAH caused a significant increase in caspase-3-positive apoptotic neurons. Taken together, our results confirmed that the SAH model induced a motor disturbance associated with late neuronal death, indicating signs of DCI, and reproducing neurological and motor symptoms found in SAH patients.

Our prospective human study revealed that various cytokines, chemokines, and growth factors varied over time. ICAM-1, bFGF, IL-7, IL-12p40, and MCP-4 variations over time were different in SAH patients with good versus bad outcomes. The increased concentration of ICAM-1 within 24 h was already known to be linked with the severity of symptoms in SAH patients [48]. bFGF was implicated in fibrosis, angiopathy, and hyperplasia during late stages in SAH patients [49]. Moreover, high levels of Flt-1 and VEGF at admission were associated with a worse outcome in SAH. Several of these factors are crucial in maintaining BBB integrity. In particular, VEGF increases BBB permeability [50], and mouse experiments have shown that anti-VEGF treatment decreases permeability and severity of symptoms [51, 52], confirming the importance of VEGF on BBB integrity and EBI. In our human samples, SAA varied significantly over time, but was not associated with the prognosis. However, SAA demonstrated a predictive value for detecting patients susceptible to developing a nosocomial infection during their hospitalization [53]. Furthermore, IL-6 is significantly increased over time, but not linked to the severity of SAH. IL-6 is considered a biomarker of vasospasm [54], a finding that was not reproduced in our study. Several markers that varied over time in our study seemed unrelated to DCI, vasospasm, or clinical outcomes in another study [55]. Therefore, further studies are required to better understand the impact of these inflammatory proteins in SAH.

Altogether, these observations suggest that alteration to the BBB may allow innate immune cell recruitment into the brain to promote intracerebral inflammation, leading to neuronal cell death and motor deficits following SAH.

The limitations in our study are primarily linked to our model. Other models exist, such as the endovascular perforation in rats, which reproduces severe SAH. It can generate an important ischemia, but at the expense of mortality [56]. Most other models were developed to study cerebral vasospasm, but have little focus on the alternative pathways of brain injury [57]. In contrast, our model mimics an anterior circulation SAH, which is clinically relevant. Prospective immunomonitoring of circulating leukocytes in SAH patients will lead us to a better understanding of cellular inflammation during EBI and will be assessed in an upcoming study.

## Conclusions

In this study, we showed that early infiltration and activation of innate immune cells (neutrophils, classical monocytes, and activated microglia and macrophages) precede late neuronal death and motor deficits associated with SAH. Macrophage reprogramming offers us a new window for pharmacological treatments in SAH. Long-term goals to improve patient outcomes are to modulate microglia activation and macrophage phenotype to decrease neuroinflammation and neuronal cell death, ultimately reducing functional deficits and improving outcomes in SAH patients.

## Data Availability

The datasets generated during and/or analyzed in the current study are available from the corresponding author on a reasonable request.

## Abbreviations

ACK: Ammonium chloride potassium
BBB: Blood brain barrier
bFGF: Basic fibroblast growth factor
CI: Confidence interval
CIPA: Comité Institutionnel de Protection des Animaux
CONSCIOUS: Clazosentan to Overcome Neurological iSChemia and Infarction OccUring after Subarachnoid hemorrhage
CRCHUM: Centre de Recherche du Centre Hospitalier l’Université de Montréal
CRP: C-reactive protein
CSF: Cerebrospinal fluid
CT: Computed tomography
D: Day
DCI: Delayed cerebral ischemia
EBI: Early brain injury
EDTA: Ethylenediaminetetraeacetic acid
EVD: External ventricular drainage
GFAP: Glial fibrillary acidic protein
GM-CSF: Granulocyte-macrophage colony stimulating factor
h: Hours
HBSS: Hank’s balanced salt solution
Iba-1: Ionized calcium-binding adapter molecule 1
ICAM-1: Intercellular adhesion molecule-1
IFN-γ: Interferon-γ
IL: Interleukin
IP: Interferon-inducible protein
MCP-1: Monocyte chemoattractant protein-1
MDC: Macrophage-derived chemokine
MIP: Macrophage inflammatory protein
mRS: Modified Rankin Scale
NeuN: Neuronal nuclei
PBS: Phosphate buffered saline
PlGF: Placental growth factor
rpm: Rotation per minutes
SAA: Serum amyloid A
SAH: Subarachnoid hemorrhage
SEM: Standard error of the mean
TARC: Thymus and activation-regulated chemokine
Tie: Tyrosine kinase with immunoglobulin and EGF homology domains
TNF: Tumor necrosis factor
VCAM: Vascular cell adhesion molecule
VEGF: Vascular endothelial growth factor
WFNS: World Federation of Neurological Surgeons
WT: Wild type
